# Life Satisfaction during Forced Social Distancing and Home Confinement Derived from the COVID-19 Pandemic in Spain

**DOI:** 10.3390/ijerph18041474

**Published:** 2021-02-04

**Authors:** Jerónimo J Gonzalez-Bernal, Paula Rodríguez-Fernández, Mirian Santamaría-Peláez, Josefa González-Santos, Benito León-del-Barco, Luis A. Minguez, Raúl Soto-Cámara

**Affiliations:** 1 Department of Health Sciences, University of Burgos, 09001 Burgos, Spain; jejavier@ubu.es (J.J.G.-B.); mspelaez@ubu.es (M.S.-P.); rscamara@ubu.es (R.S.-C.); 2 Department of Psychology, Faculty of Teacher Training College, University of Extremadura, 10071 Caceres, Spain; bleon@unex.es; 3 Department of Educational Sciences, University of Burgos, 09001 Burgos, Spain

**Keywords:** life satisfaction, well-being, pandemic, COVID-19, forced social distancing, home confinement

## Abstract

Life satisfaction is one of the main dimensions of well-being related to psychological factors, being essential for a person to adjust to difficult circumstances. The restrictive measures adopted to minimize the diffusion of the coronavirus disease (COVID-19) could alter the subjective dimension of well-being, so the objective of this study was to determine the factors related to life satisfaction of the Spanish population during forced home confinement derived from the COVID-19 pandemic. A cross-sectional study was designed, based on an online survey, and disseminated through the main social networks, which included the Satisfaction with Life Scale (SWLS) and sociodemographic and COVID-19-related variables. The possible relationships between the different variables were studied using univariate and multivariable regression analyses. A total of 3261 subjects participated in the study. Factors associated with greater personal life satisfaction were fewer days of home confinement (β = (−0.088); *p* ≤ 0.001), the perception of having received enough information (β = 0.076; *p* ≤ 0.001), having private access to the outside (β = 0.066; *p* ≤ 0.001), being employed (β = 0.063; *p* ≤ 0.001), being male (β = 0.057; *p* = 0.001) and not having been isolated (β = 0.043; *p* = 0.013). The results of this study provide novel information about the profiles of people related to greater well-being and life satisfaction during forced social distancing and home confinement, but more studies are needed to help to understand and complement these findings.

## 1. Introduction

In December 2019, an outbreak of severe acute respiratory syndrome associated with a new coronavirus, SARS-CoV-2, was identified for the first time in the Chinese city of Wuhan. In the following weeks, this atypical pneumonia, named coronavirus disease-19 (COVID-19), attracted the attention of the Health Authorities for its unpredictable, rapid and exponential spreading, with a high mortality rate [1]. This new disease has posed serious challenges to global health [2], forcing the World Health Organization (WHO) to recognize it as the sixth public health emergency of international importance on 30 January 2020 and declare the situation a global pandemic on 11 March 2020 [3]. As a consequence, many countries decided to adopt very restrictive measures to minimize the diffusion of the SARS-CoV-2. Specifically in Spain, one of the most affected territories, on 14 March 2020, forced social distancing and compulsory home confinement were announced, and free movement was restricted to avoid the collapse of the health system [4].

The implementation of a mandatory quarantine period along with other public health measures has proven effective in slowing down the transmission of the virus. These measures imply a radical change in the lifestyle of the person and can lead to a perception of restricted freedom and increase the prevalence of risk behaviors for health [5,6,7,8,9].

The progression and contagion of emotional patterns of indifference, curiosity and fear during forced home confinement are related to the development and establishment of responsible behavior patterns among citizens. Individuals redefine social reality in cognitive–emotional terms to face fear and uncertainty of the unknown, developing social patterns of behavior that emphasize co-responsibility, solidarity and civic culture [10].

Isolation and loneliness are psychosocial risk factors that, together with the fear of contracting the disease and exacerbation of interpersonal problems during forced social distancing and home confinement, can trigger serious public mental health problems [6,9,11]. Forced social distancing and home confinement, in addition to reducing a person’s participation in community and family life [12], have had harmful psychological effects even on uninfected people, since uncertainty and fear of the unknown can have very negative consequences on people’s health and lives [13,14]. Meta-analytic evidence suggests that situations of social isolation affect longevity in the same way or more than somatic factors such as smoking, alcohol consumption, sedentary lifestyle or obesity [15]; and people integrated into the community and with frequent social relationships are less likely to see their quality of life and well-being affected by stressful events [16,17,18,19].

Findings from different studies have reported the psychosocial impact of the pandemic at the level of individual well-being of the person, in form of psychological symptoms such as emotional disorders, depression, stress, irritability, insomnia, post-traumatic stress, anger or emotional exhaustion [12,20]; risk behaviors such as increased substance abuse [21]; or decreased perceived health [22]. However, these responses also affect the level of community well-being [23,24,25]. The WHO recommends paying special attention to social participation, which plays an important role in the different dimensions of well-being [26], especially in the life satisfaction [27,28], defined as the estimation of quality of life based on the preferences and complacency of an individual with respect to the different dimensions of their life [29]. This is a fundamental aspect of social well-being [29,30,31,32] and is related to important psychological variables [33]. Numerous studies have reported that a greater life satisfaction is associated with better mental and physical health and well-being and with better cognitive and social functioning [34]. Thus, a good life satisfaction is essential for a person to adjust to difficult circumstances, and even to benefit from them [21,23,29,31].

In recent months, most efforts have been directed at controlling the spread of the virus and seeking an effective vaccine and/or treatment for infected people, leaving the psychosocial aspect of the pandemic unattended [6,13,35]. The different proactive and preventive measures implemented to manage the spread of the pandemic in Spain may be causing an unprecedented alteration in the well-being of the population. For this reason, special attention must be paid to aspects related to life satisfaction, essential in times of crisis, which allows the person to adapt to complicated situations or even come out of them reinforced. In order to establish support programs for people during forced social distancing and home confinement, it has been necessary to analyze the impact of different factors on life satisfaction. These data can provide significant information related to the profiles most vulnerable or resistant to suffering alterations in this dimension of well-being. From all this, the existence of a relationship between sociodemographic and COVID-related variables and life satisfaction in the Spanish population during home confinement was proposed as a hypothesis to be tested. Therefore, the main objective was to determine the factors related to life satisfaction of the Spanish population during forced home confinement derived from the COVID-19 pandemic.

## 2. Materials and Methods

### 2.1. Study Design—Participants

A cross-sectional study was designed, whose study population consisted of all people aged over 18 years residing in Spain during the forced home confinement phase produced as a consequence of the COVID-19 pandemic.

### 2.2. Procedure—Data Collection 

Based on the voluntary character of the study, participants were selected by a non-probabilistic convenience sampling. Given the circumstances of forced social distancing and home confinement in which the study was carried out, using an online survey through Google Forms, disseminated across the main social networks such as Facebook, Twitter or WhatsApp, was considered the best data collection strategy to access the maximum number of people and information. The participants were able to access and complete the questionnaire from 16 March 2020 to 10 May 2020, the time interval between the first working day of forced home confinement and the first working day on which the forced home confinement rules were relaxed. In the first part of the survey, a short presentation informed the participants about the main objective of the study, requesting their anonymous and voluntary participation. In order to guarantee the anonymity of the participants, no personal data which could allow their identification were collected. The individual informed consent to participate in the study was implicit in the completed return of the survey. Those questionnaires which were not fully completed were eliminated from subsequent analysis. Each participant had to answer the questionnaire twice at the same time, and the questionnaire collected sociodemographic and COVID-19-related variables as well as information about life satisfaction. In the first occasion, responses had to refer to the week immediately prior to forced home confinement, while in the second one, to the time of survey completion. The estimated time needed for filling in the survey was 15–20 min. 

The study was conducted according to the guidelines of the Declaration of Helsinki and received a favorable report from the Institutional Review Board of the University of Burgos (Protocol code 14/2020—April 2020).

### 2.3. Main Outcomes—Instruments

The main outcome of the study was the differential score between satisfaction with life the week immediately prior to the forced home confinement and at the time of the survey completion measured through the Satisfaction with Life Scale (SWLS) [34]. The SWLS is a self-administered questionnaire, designed by Diener et al. in 1985 [34], and later reviewed by Pavot and Diener in 2009 [36], which evaluates the global cognitive judgments of one’s life satisfaction. The Spanish version, adapted and validated by Vazquez et al. in 2000 [37], is a valid and reliable measure of life satisfaction and includes 5 items, in which each participant must indicate their degree of agreement with the content of the sentence using a five-point Likert scale, where 1 corresponds to “completely disagree” and 5 to “totally agree”. The total score can range from 5 to 25, with higher scores indicating greater life satisfaction [38]. Confirmatory factor analysis reveals a unifactorial structure with significant correlations between the SWLS and subjective happiness and social support. The internal consistency of this scale was 0.88 [37].

Sociodemographic and COVID-19-related information was also collected such as age, sex, marital status, employment status, educational level, residence area, number of rooms at home, possibility of private access abroad, number of people who make up the family nucleus, number of children under 18 years or dependent at home, days of confinement at the time of completing the questionnaire, suffering or having suffered from COVID-19, having required home isolation, or perception of the information received. For this, an ad hoc questionnaire was designed, which was previously piloted in a sample of 15 people who were not part of the subsequent analysis.

### 2.4. Statistical Analysis

In order to characterize the sample, descriptive analyses were conducted. Absolute frequencies and percentages were used if the variables were categorical or mean and deviation (SD) in case of continuous variables. The compliance of the normality criteria of the quantitative variables was assessed by Kolmogorov–Smirnov test. To determine the existence of association between the variation in the life satisfaction and the categorical variables, the independent sample Student’s t test was used. The effect size differences were calculated using the Hedge’s g and interpreted according to the following criteria: If 0 ≤ g < 0.01, there is no effect; if 0.01 ≤ g < 0.06, the effect is minimal; if 0.06 ≤ g < 0.14, the effect is moderate; and if g ≥ 0.14, the effect is strong. To evaluate the relationship between the differential score obtained in the SWLS and the quantitative variables, the Pearson correlation was used. To quantify the magnitude of these associations and identify possible independent predictive factors related to greater life satisfaction during home confinement derived from COVID-19 infection, a forward stepwise multiple lineal regression analysis, adjusted by sex and age, was performed. In this model, all variables with a *p*-value < 0.05 in the univariate analysis were included. Statistical analysis was performed with SPSS version 25 software (IBM-Inc, Chicago, IL, USA). For the analysis of statistical significance, a value of *p* < 0.05 was established.

## 3. Results

The number of subjects who voluntarily agreed to participate in the study was 3261. Overall, 81.69% (*n* = 2664) of these participants were women. The age of the participants ranged from 18 to 93, with a mean of 40.53 years (SD ± 14.05). Of the 1891 participants who were previously working, 58.12% (*n* = 1099) continued working or telecommuting during the home confinement phase. Regarding households, the mean number of people who made up the family nucleus was 2.96 (DE ±1.23), with children under 18 years or dependent at home in 30.54% and 14.35% of the cases, respectively. At the time of completing the survey, 3.25% (*n* = 106) of participants reported that they had suffered or were currently suffering from SARS-CoV-2 infection, and 4.60% (*n* = 150) that they had required isolation. The perception of having received insufficient information about the infection by COVID-19 was generalized, with 2043 persons reporting it. The confinement time elapsed at the moment of completion of the questionnaire ranged from 4 to 57 days. The mean differential score obtained in the SWLS was (−2.77) (SD ± 4.09). 

When comparing the differential scores of the SWLS with the sociodemographic and COVID-19-related categorical variables, being male (*p* = 0.002), being employed (*p* < 0.001), having a higher educational level (*p* = 0.003), having private access to the outside during forced confinement at home (*p* < 0.001), not having been isolated at home at the time of filling in the form (*p* = 0.038) and the perception of having received enough information regarding the COVID-19 pandemic (*p* < 0.001) were associated with a higher life satisfaction. In order to complete the information provided by the statistical significance tests and to determine between which variables the differences were more intense, the effect size was calculated using the Hedge´s g statistic, since a “non-significant” result may have practical significance. Perception of the information received, isolation, employment status and educational level showed a strong effect size on the differential score of life satisfaction (Table 1). The internal consistency of this scale was 0.87.

Likewise, the correlations between the differential scores of life satisfaction and sociodemographic and COVID-19-related continuous variables are shown in Table 2. In this sense, a statistically significant weak and negative correlation with the number of days of home confinement (*p* < 0.001) and the number of people who lived together at home (*p* = 0.032) was observed, while a statistically significant weakly and positively correlation between with the number of rooms at home (*p* = 0.029) and the number of children under 18 years at home (*p* = 0.029) was obtained. No correlation was observed between the differential scores of SWLS and age (*p* = 0.354) and the number of dependents at home (*p* = 0.897).

In the multivariate analysis, independent factors related to greater personal life satisfaction were the number of days of home confinement, the perception of having received enough information, having private access to the outside, being employed, being male and not having been isolated (Table 3). Other factors included in this regression model were not significantly associated with life satisfaction. The variables included in the regression model explained 3% of the variation in the differential score of life satisfaction during forced home confinement (R² = 0.030, F_(6, 3254)_ = 16.855, *p* < 0.001). 

## 4. Discussion

Forced social distancing and home confinement have been the most restrictive measures to contain the diffusion of SARS-CoV-2 in the Spanish population. Understanding the psychosocial implications of these measures could facilitate pandemic management and promote better-informed decision-making. In this study, designed to provide an accurate picture of the life satisfaction of Spanish people during the period of forced social distancing and home confinement, being male, being employed, having private access to the outside, fewer days of home confinement, the perception of having received enough information and not having been isolated were independent predictive factors of a higher life satisfaction.

Findings of several studies in previous health crises, such as the severe acute respiratory syndrome outbreak caused by SARS-CoV in 2002–2004, have revealed a wide range of negative aspects of restrictive measures on individual well-being [39,40] and quality of life [40]. More recent studies have analyzed the psychological effect and the consequences of social distancing and home confinement derived from COVID-19 in different countries of the world. Decreased social participation, changes in relationships with family and friends and isolation have been associated with feelings of loneliness, pain and loss of quality of life [12,20,41]. During quarantine periods, physical activity is also reduced, and the consumption of unhealthy foods increases, decreasing the well-being of the population [42]. People subjected to forced home confinement have reported greater symptoms of psychological distress and lower life satisfaction [43], which persist long after the quarantine period in most cases [25]. There is no clear evidence about the predictor factors of the psychological impact of quarantine. The results of a study carried out on the Portuguese and Brazilian populations suggested that sociodemographic factors such as being female, having a higher educational level, being a student, living with family members or a partner in the period of social isolation or low levels of depression were significantly associated with increased levels of life satisfaction scores [44]. However, in another study, age, marital status, educational level, co-living status, or having children were not significantly associated with this psychological data [45]. 

The fear of being infected by SARS-CoV-2 along with the stress produced by the restrictive isolation measures have also been negatively related to people’s life satisfaction [46,47,48]. From this perspective, the results of this study are similar to those obtained by Brooks et al. [11], who demonstrated that long periods of quarantine, economic losses and perception of having received deficient information from Public Health Authorities were some of the stressors during home confinement and, therefore, one of the main causes of decreased life satisfaction. On the other hand, Li et al. [49] analyzed the relationship between self-compassion and life satisfaction in the Chinese population during the quarantine period derived from the COVID-19 pandemic, showing that the life satisfaction of women with high levels of positive coping was not higher than that obtained from men. These findings are in line with those observed in the present study, in which being a man is a protective factor of life satisfaction.

As in the studies mentioned above, being employed contributed to a greater life satisfaction. During home confinement, a large part of the population was forced to stop working, which meant an abrupt interruption of economic income in the majority of cases. Taking into account data from previous epidemics, financial loss resulting from a quarantine period leads to severe socioeconomic distress and triggers symptoms of psychological distress and alterations in quality of life [50,51]. In relation to the data received by Public Health Authorities, the perception of lack of information has been considered one of the main triggers of stress in the time of a pandemic [52,53,54,55]. Keeping public media in operation and not taking any action that hinders social interaction beyond the need to keep physical distance is essential for the well-being of society [56]. Likewise, a clear and transparent communication by the authorities when issuing new instructions about the different measures to stop the spread of COVID-19 is essential to achieve compliance with the regulations and avoid the dynamics of collective change [56,57]. The absence of clear guidelines on actions to take and the confusion about the purpose of quarantine have significantly influenced people’s mental health and life satisfaction [52,53], since in situations of existential uncertainty, most people redouble their efforts to preserve a shared and coherent vision of social reality [58]. A cross-sectional study carried out in Vietnam by Nguyen et al. [27] demonstrated that health literacy is a protective factor for depression and life satisfaction during the current pandemic. All these findings reaffirm those results obtained in this study, where the perception of sufficient information received has proven to be one of the main contributing factors to greater life satisfaction during forced home confinement.

This study provides information about the life satisfaction of the Spanish population during the forced home confinement derived from the COVID-19 pandemic. As practical implications, the risk and protective factors associated with life satisfaction in periods of social isolation provide data for the enactment of policies that attempt to lessen the impacts of the COVID-19 pandemic on well-being. Greater attention and assistance should be prioritized for the most vulnerable groups of the population, such as the female gender, people without employment, people without private access to the outside and those who are subjected to longer quarantine periods. Adequate and sufficient information has proven to be a fundamental protective factor for life satisfaction and well-being in times of crisis, so governments must ensure proper and timely dissemination of information related to COVID-19. However, findings should be interpreted considering their limitations and strength. Using online surveys has been one of the best strategies to collect data during forced home confinement, although these tools could have led to a bias in recruitment of participants and influenced the characteristics of the sample, such as the high number of women. Furthermore, this data collection option allows assessing life satisfaction of the participants prior to the forced social distancing phase, avoiding their loss during the follow-up period. Despite the high sample size of the study, it has not been possible to determine the causality inferences between variables due to the cross-sectional nature of the study. The use of a convenience sample may have induced a higher response rate from people who were very interested in the issue. To evaluate life satisfaction prior to the forced home confinement, the participants had to respond to the questionnaires on a single occasion, but referring to two different moments in times, which may have influenced the obtained responses. Regarding multiple regression analysis, the determination coefficient (R^2^) obtained in the model only explains 3% of the variance of life satisfaction. Cohen maintains that a magnitude of R^2^ equal to or less than 0.02 indicates a small effect size [59]. Other authors state that this value is not too important with samples greater than 100, even less so if the model has more than 5 predictors, since it is the low *p*-values that indicate a real relationship between the significant predictive factors and the response variable [60]. Regarding the discussion of the obtained results, the lack of specific studies on this topic has made it difficult to contrast and compare them. All of these limitations may have influenced the obtained results and reduced their generalizability to the general population. The strengths of this study include the collection of data on a large sample of Spanish adults and the multivariate analysis of a wide and varied set of variables.

When it is necessary to again implement restrictive measures to contain the diffusion of SARS-CoV-2, the results obtained in this study could be considered, ensuring better management of the pandemic and thus achieving greater well-being and quality of life in the general population. More research is needed to help to understand and analyze the effects of the COVID-19 pandemic on people’s life satisfaction and mental health and to achieve the maximum well-being of the population in times of crisis.

## 5. Conclusions

Life satisfaction decreased as the days of home confinement progressed. Different sociodemographic and COVID-19-related variables contributed to greater life satisfaction during the most restrictive quarantine period experienced to date in Spain. Being a man, being employed, having private access to the outside, the perception of having received enough information about the pandemic and not having been isolated promoted greater life satisfaction during the forced social distancing and home confinement.

## Figures and Tables

**Table 1:** Comparison of differential score in the Satisfaction with Life Scale (SWLS) according to categorical variables.

	*n* (%)	Differential Score in SWLS		*p*-Value	Hedge’s g
		Mean	SD		
**Sex**					
Female	2664 (81.69%)	(−2.87)	4.18	0.002	0.132
Male	597 (18.31%)	(−2.33)	3.67		
**Marital status**					
Single/Separated/Divorced/Widower	1221 (37.44%)	(−2.76)	(4.09)	0.908	0.002
Married/Living with a partner	2040 (62.56%)	(−2.77)	(4.10)		
**Educational level**					
No formal studies/Primary or secondary studies	648 (19.87%)	(−3.24)	4.67	0.003	0.144
Vocational training studies/University studies	2613 (80.13%)	(−2.65)	3.93		
**Employment status**					
Unemployed/Home chores/Student/Retired	1370 (42.01%)	(−3.18)	4.42	<0.001	0.171
Active	1891 (57.99%)	(−2.48)	3.82		
**Residence area**					
Urban	2271 (69.64%)	(−2.79)	(4.04)	0.653	0.017
Rural	990 (30.36%)	(−2.72)	(4.21)		
**Private access to the outside**					
No	1216 (37.29%)	(−3.11)	4.38	<0.001	0.132
Yes	2045 (62.71%)	(−2.57)	3.90		
**Currently infected by SARS-CoV-2**					
No	3223 (98.83%)	(−2.75)	4.08	0.060	0.381
Yes	38 (1.17%)	(−4.31)	4.95		
**Infected by SARS-CoV-2**					
No	3193 (97.91%)	(−2.75)	4.08	0.114	0.213
Yes	68 (2.09%)	(−3.62)	4.43		
**Isolation**					
No	3111 (95.40%)	(−2.73)	4.08	0.038	0.183
Yes	150 (4.60%)	(−3.48)	4.27		
**Perception of the information received**					
Insufficient	2043 (62.65%)	(−3.05)	4.31	<0.001	0.184
Enough	1218 (37.35%)	(−2.30)	3.66		

*n*: Number of participants; SWLS: Satisfaction with Life Scale; SD: Standard deviation. The bold indicates the tittle of each of the variables.

**Table 2:** Correlation between differential scores in the SWLS and quantitative variables.

	Mean	SD	r Pearson with Differential Score in SWLS	*p*-Value
Age	40.53	14.05	0.016	0.354
Days of home confinement	22.69	13.30	(−0.103)	<0.001
Number of people at home	2.96	1.23	(−0.037)	0.032
Number of children under 18 years at home	0.49	0.87	0.038	0.029
Number of dependents at home	0.18	0.50	0.002	0.897
Number of rooms at home	5.27	2.17	0.038	0.031

SWLS: Satisfaction with Life Scale; SD: Standard deviation.

**Table 3:** Multiple linear regression analysis of independent predictive factors for a higher life satisfaction.

Independent Predictive Factors	Standard Error	β	t	*p*-Value
Days of home confinement	0.006	(−0.088)	−4.889	<0.001
Perception of received enough information	0.147	0.076	4.390	<0.001
Having private access to the outside	0.146	0.066	3.847	<0.001
Being employed	0.149	0.063	3.535	<0.001
Sex: Male	0.184	0.057	3.292	0.001
Not staying isolated	0.338	0.043	2.488	0.013
